# ASO Author Reflections: Real-World Effectiveness of Prehabilitation Before Colorectal Cancer Surgery: The Value of an Emulated Target Trial Design

**DOI:** 10.1245/s10434-022-12672-0

**Published:** 2022-10-17

**Authors:** Thea C. Heil, Emiel G. G. Verdaasdonk, Huub A. A. M. Maas, Barbara C. van Munster, Marcel G. M. Olde Rikkert, Johannes H. W. de Wilt, René J. F. Melis

**Affiliations:** 1 Department of Geriatric Medicine, Radboud University Medical Center, Nijmegen, The Netherlands; 2 Department of Surgery, Jeroen Bosch Hospital, ‘s-Hertogenbosch, The Netherlands; 3 Department of Geriatric Medicine, Elisabeth-Tweesteden Hospital, Tilburg, The Netherlands; 4 Department of Internal Medicine, University Medical Center Groningen, University of Groningen, Groningen, The Netherlands; 5 Department of Surgery, Radboud University Medical Center, Nijmegen, The Netherlands

## Past

While the prehabilitation concept appears promising, there is conflicting scientific evidence for the effectiveness.^1^ Several RCTs have been performed showing different effects of multimodal prehabilitation, ranging from meaningful changes in postoperative functional walking capacity and significantly improved postoperative clinical outcomes to no effect on postoperative outcomes.^2, 3^ Because these RCTs are usually performed under ideal circumstances, including only a selected group of patients, this can compromise external validity.^2–4^ Studies based on observational, real-world data could be of added value to better understand for whom prehabilitation may be of benefit. The purpose of this study was to evaluate the effect of a multimodal prehabilitation program compared with usual care on perioperative outcomes in patients undergoing elective colorectal surgery with a higher postoperative complication risk, using an emulated target trial (ETT) design.

## Present

This is the first, single-center ETT to assess the impact of a prehabilitation intervention on perioperative complications in patients aged ≥65 years or <65 years and ASA III/IV, undergoing colorectal cancer surgery.^5^ The ETT included 251 patients: 128 in the usual care group and 123 patients in the prehabilitation group. In the intention-to-treat analysis, the number needed to treat to reduce one or more complications in one person was 4.2 (95% confidence interval [CI] 2.6–10). Compared with patients in the usual care group, patients undergoing prehabilitation had a 55% lower comprehensive complication score (95% CI−71% to−32%). There was a 33% reduction (95% CI −44% to −18%) in LOS from 7 to 5 days. Prehabilitation had no effect on the already limited number of readmissions. Additional to previous RCTs that showed positive evidence on the effectiveness of prehabilitation, this study provides the first preliminary evidence that prehabilitation does not only improve outcomes in a controlled study setting but also in daily clinical practice.

## Future

The methodology used (ETT) in this study could be an example for further multicenter studies. However, to be able to use (large) clinical registration databases in future ETTs on the effect of prehabilitation and other complex interventions, these databases should incorporate more clinical predictor variables (e.g., frailty score), further specification of clinical outcomes (e.g., CCS-score), and patient-reported outcome measures (e.g., quality of life).
